# A Fatal Case of Disseminated Histoplasmosis by *Histoplasma capsulatum* var. *capsulatum* Misdiagnosed as Visceral Leishmaniasis—Molecular Diagnosis and Identification

**DOI:** 10.3390/pathogens12091112

**Published:** 2023-08-30

**Authors:** Manuel Calvopiña, Marcelo Toro, Carlos Bastidas-Caldes, David Vasco-Julio, Greta Muñoz

**Affiliations:** 1 One Health Research Group, Universidad de Las Américas (UDLA), Quito 170124, Ecuador; carlos.bastidas@udla.edu.ec; 2 Hospital Pediátrico “Baca Ortiz”, Quito 170523, Ecuador; marcelo.toro@hbo.gob.ec (M.T.); greta.munoz@hbo.gob.ec (G.M.); 3 Programa de Posgrado en Ciencias Biológicas, Universidad Nacional Autónoma de México, Mexico City 04510, Mexico; david.vasco.julio@outlook.com; 4 Centro de Investigación Sobre Enfermedades Infecciosas, Instituto Nacional de Salud Pública, Cuernavaca 62050, Mexico

**Keywords:** histoplasmosis, *Histoplasma capsulatum*, molecular diagnosis, Ecuador

## Abstract

Histoplasmosis is an endemic mycosis in the Americas. However, its diagnosis is challenging due to the complexity and limited availability of conventional laboratory techniques—antigen tests, culture, and staining. Microscopic preparations often confuse with other pathogens, such as *Leishmania* spp. The genus *Histoplasma capsulatum* comprises three varieties: var. *capsulatum*, var. *duboissi*, and var. *farciminosum*, which cannot be distinguished using conventional techniques. An infant from a tropical region of Ecuador was hospitalized for fever, bloody diarrhea, and anemia persisting for two months. Upon admission, he received antibiotics and immunosuppressants. Histopathological examination of the lymph nodes, intestines, and bone marrow aspirate reported the presence of *Leishmania*-like amastigotes, and treatment was initiated with meglumine antimoniate and conventional amphotericin B. However, subsequent analysis of samples using PCR and DNA sequencing identified *H. capsulatum* var. *capsulatum* but not *Leishmania*. Despite fluconazole and amphotericin B, the infant succumbed to the disease. The delay in clinical and laboratory diagnosis of histoplasmosis and the use of nonspecific and ineffective drugs such as fluconazole led to disease dissemination and, ultimately, death. Implementing molecular diagnosis and antigen tests in laboratories located in endemic regions and reference hospitals is crucial.

## 1. Introduction

Histoplasmosis is a fungal infection caused by inhaling spores belonging to the genus *Histoplasma capsulatum*, commonly found in the droppings of bats and birds. It is documented worldwide, except in Antarctica, and is endemic and prevalent in tropical regions of Latin American countries [1,2]. *H. capsulatum* is traditionally classified into three varieties: *H. capsulatum* var. *capsulatum*, which is distributed worldwide and causes various clinical manifestations; *H. capsulatum* var. *duboisii*, found in Africa and associated with aggressive disease; and *H. capsulatum* var. *farciminosum*, reported as a pathogen in horses in Asia [1,3]. Discrimination among these three varieties is achieved through nucleotide amplification assays targeting multicopy genes, as the ribosomal DNA 18S, 28S, and 5.8S genes and the internal transcribed spacer (ITS) region, derived from various fluids and tissues [4]. These fungal isolates have recently been reclassified into different phylogenetic species [5,6,7]. For instance, *H. capsulatum* isolates from the Americas have been subdivided into four distinct clades. However, these clades/species designations do not strictly adhere to the International Code of Botanical Nomenclature rules and are considered invalid [1].

Ecuador is recognized as an endemic country for *H. capsulatum*, with the first documented case dating back to 1953 [8]. Since then, several cases have been reported, demonstrating the prevalence of pulmonary, liver, skin, bone marrow, intestinal, and disseminated infections [9,10,11,12], been diagnosed in patients with HIV/AIDS [13,14,15,16]. Ecuadorians had been diagnosed in European countries [17,18,19], and in Chilean and European tourists visiting Ecuador [20,21]. Additionally, cases reported in nontropical areas [22]. Official data indicate 232 cases in the past 10 years [23]. In Ecuador, the varieties of *H. capsulatum* have not been distinguished, and all infections are reported as *H. capsulatum*.

The clinical presentation of histoplasmosis varies depending on the site of infection, age, degree of immunosuppression, size of the inoculum, and the infecting variety. Most cases (around 90%) are asymptomatic or exhibit self-limited symptoms. In HIV patients with low CD4 counts and individuals receiving immunosuppressive medications, including glucocorticoids, and in inhered immunodepression, the risk of severe disseminated infections and death is increased [24]. This risk is also elevated in malnourished children [25]. Gastrointestinal involvement is common in histoplasmosis, leading to symptoms such as pain, bleeding, perforation, and malabsorption; this manifestation can present as mass lesions or ulcerations [26].

The pharmacological management of histoplasmosis depends on the severity and progression of the disease. Mild cases may not require antifungal treatment unless the patient is immunosuppressed, or symptoms persist for more than four weeks. Itraconazole is the preferred treatment in moderate cases, with voriconazole and posaconazole as additional options. Fluconazole is not effective. For severe and disseminated cases, liposomal amphotericin B is the treatment of choice [27].

Microscopic observation of *H. capsulatum* yeasts and isolation in specific cultures remain the “gold standard” for diagnosis [24,28]. Recommended stains for different cytological and histopathological preparations include Grocott’s methenamine silver (GMS) and periodic acid–Schiff (PAS) [28]. In specific culture media, *H. capsulatum* requires a biosafety level 3 laboratory and typically takes 2 to 4 weeks, or up to 8 weeks, to grow [28]. Histopathological and cytological examinations of peripheral blood smears, bone marrow, bronchoalveolar lavage fluid, or fine-needle aspiration of tissues stained with hematoxylin and eosin (H&E) reveal yeasts consistent with *H. capsulatum* [28,29]. Other pathogens that need to be differentiated by microscopy include amastigotes of *Leishmania* spp., *Trypanosoma cruzi*, *Toxoplasma gondii*, and other fungi. These protozoa do not stain with GMS and PAS but can be visualized using H&E and Giemsa staining [28]. Leishmaniases, Chagas disease, and toxoplasmosis are also endemic in tropical regions [30]. Antigen tests for *H. capsulatum* have high sensitivity in blood and urine [31]. However, cross-reactions with other endemic mycoses, such as paracoccidioidomycosis, have been reported, and antigen tests may not be readily available in endemic regions [26,27,28,31]. Antibody detection, although sensitive and easy to interpret, exhibits cross-reactivity with other endemic mycoses and is detectable 4 to 8 weeks after the initial infection [24]. Molecular diagnostic techniques have recently been developed for the direct detection and sequencing of *Histoplasma* spp. DNA. These techniques can be performed on various clinical specimens such as blood, serum, urine, biopsies, aspirates, and cultures [29]. Several molecular targets are utilized, most employing PCR-amplification of the 18S rDNA gene, the 100 kDa protein (Hcp 100), and the M and H proteins. Amplification and sequencing of the internal transcribed spacer (ITS) regions 1 and 2 and the 5.8S region have been employed to detect and characterize *Histoplasma* species [24,32].

In this context, we present a case of an infant from a tropical region of Ecuador who was admitted with symptoms of fever, bloody diarrhea, and anemia. Microscopic examination wrongly reported *Leishmania* amastigotes, intracellular structures that actually were *H. capsulatum* yeasts, and the patient was treated with meglumine antimonate followed by conventional amphotericin B, based on this finding. However, subsequent PCR and Sanger sequencing analysis identified *H. capsulatum* var. *capsulatum*. The infant, unfortunately passed away despite treatment.

## 2. Case Report

A 2-year-old boy residing in the tropical city of Esmeraldas, Ecuador, was transferred from Esmeraldas Hospital to the Pediatric Hospital in Quito due to a two-month history of fever, bloody diarrhea, and anemia. He had received treatment from a private doctor and at Esmeraldas Hospital, including blood transfusions, antipyretics, antidiarrheals, and various antibiotics such as amikacin, trimethoprim-sulfamethoxazole, ampicillin-sulbactam, and ceftriaxone. The patient also had a history of controlled atopic dermatitis managed with emollients and topical corticosteroids.

Upon admission, the patient was febrile (38.5 °C) with a heart rate of 130 beats per minute, respiratory rate of 28 breaths per minute, and oxygen saturation of 90%. The patient’s weight was 8.6 kg (z score −4.18), height was 75 cm (z score −4.27), and weight-for-height was −1.54 z score. The body mass index was 15.3 (z score −0.94). The patient was active and irritable to handling, hydrated, and showed no signs of respiratory distress. During the physical examination, whitish plaques surrounded by an erythematous halo were observed on the buccal and pharyngeal mucosa. There were no adenopathies in the neck, and the chest exhibited preserved expansibility with no abnormalities in the heart or lungs. The abdomen was soft and depressible, deep palpation caused pain; air–fluid sounds were present, and no visceromegaly or inguinal lymphadenopathy were detected. The patient had dry, scaly skin with scattered erythematous and hypopigmented areas throughout the body. The first toenail of the right foot displayed scaly hyperpigmented lesions. The remainder of the physical examination revealed no abnormalities.

During hospitalization, the patient underwent several laboratory tests, yielding the following results: leukocytes 11.1 × 10^3^/µL (reference range: 4.4–11.0 × 10^3^/µL), neutrophils 7.24 × 10^3^/µL (2.5–7.5 × 10^3^/µL), lymphocytes 3.1 × 10^3^/µL (3.0–9.5 × 10^3^/µL), hemoglobin 9.7 g/dL (9.5–13.0 g/dL), hematocrit 29.6% (30–44%), platelets 706,000/µL (150,000–450,000/µL). Additionally, the results included glucose 102 mg/dL (100–180 mg/dL), urea 11.8 mg/dL (5–18 mg/dL), creatinine 0.17 mg/dL (0.3–0.7 mg/dL), TGO 25.6 U/L (0–37 U/L), TGP 28.6 U/L (>60 U/L), GGT 22 U/L (11–50 U/L), total bilirubin 0.10 mg/dL (1 mg/dL), direct bilirubin 0.04 mg/dL (0.3 mg/dL), alkaline phosphatase 179 U/L (up to 350 U/L), LDH 203 U/L (170–580 U/L), total protein 6.5 g/dL (4.4–5.4 g/dL), albumin 2.88 g/dL (6.2–8.0 g/dL), C-reactive protein 1.33 mg/L (up to 10 mg/L), total calcium 8.96 mg/dL (4.6 mg/dL), phosphorus 3.95 mg/dL (4.0–7.0 mg/dL), sodium 138 mEq/L (136–145 mEq/L), potassium 4.68 mEq/L (3.5–5.1 mEq/L), chlorine 101 mEq/L (15–40 mEq/L), and INR 1.01 (equals 1). Stool examination revealed no parasites, 80% polymorphonuclear cells, positive occult blood, and negative stool culture.

Due to whitish lesions in the oropharynx, an upper gastrointestinal endoscopy was performed, observing whitish plaques with an erythematous border in the esophagus. The diagnosis of panesophagitis due to *Candida* spp. and grade 2 duodenitis was made, and treatment with fluconazole and oral nystatin was prescribed for 21 days. Additionally, the patient tested positive for *Helicobacter pylori*, and clarithromycin and omeprazole were added to the treatment regimen for 14 days. A blood culture showed the presence of Gram-positive cocci, leading to the administration of vancomycin for 10 days. Skin histopathology reported nonspecific dermatitis and tested negative for leprosy. Nail KOH confirmed the presence of a fungal infection, and terbinafine was prescribed for 30 days. The HIV test was negative. Chest radiography in the anteroposterior and lateral views showed no abnormalities. Abdominal ultrasonography revealed a liver with a right lobe measuring 9.5 cm (within the normal range for age). No bile duct dilation or acalculous gallbladder was observed, the pancreas and kidneys appeared normal. The spleen measured 5.7 cm and exhibited homogeneous echotexture, and no free fluid was detected in the abdominal cavity. During colonoscopy, generalized inflammation with ulcers was observed, and enteroechography findings were consistent with Crohn’s inflammatory bowel disease initiating prednisone and mesalazine but developed tachycardia after 3 days, with the discontinuation of both medications. Subsequently, methylprednisolone and azathioprine were administered for 8 days. A contrast-enhanced computed tomography of the abdomen and pelvis revealed two conglomerates of lymph nodes in the mesogastrium and multiple lymph nodes throughout the abdominal-pelvic cavity, measuring between 5 and 10 mm. The cecum and ascending colon showed a wall thickening of 18 mm, leading to luminal obliteration. The walls of the rectum, sigma, and descending colon appeared irregular and thickened.

**Figure 1:**
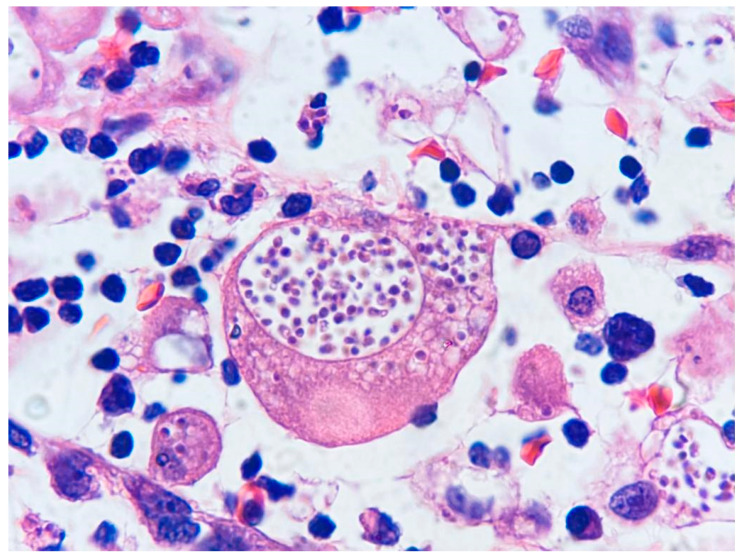
The present image from a histological section of the intestinal mucosa wrongly reported the presence of *Leishmania* amastigotes. The characteristic kinetoplasts of *Leishmania*, which typically exhibit stronger staining than the nucleus, are not observed in these oval structures. Under hematoxylin and eosin (H&E) staining at 100× magnification, the ovoid yeasts of *H. capsulatum* are visible. Yeasts measure 2 to 4 μm, have unstained cell walls with a distinct refractile capsule, and exhibit narrow-based budding. They are predominantly found phagocytosed within macrophages and histiocytes, often forming clusters of numerous organisms. Occasionally, they may also be observed in extracellular spaces [28].

After a 2-month hospitalization and persistent enterorrhagia and fever, accompanied by abdominal distension, an ultrasound examination revealed laminar free fluid in Morrison’s space and smaller volumes in the parietocolic areas (25 mL) along with pneumoperitoneum, indicating suspected intestinal perforation, metronidazole was administered intravenously for 7 days; the pediatric surgeon recommended exploratory abdominal surgery. The patient underwent partial resection of the terminal ileum due to necrosis and an enlarged lymph node extraction. Histopathology of the intestinal mucosa reported severe mixed inflammatory infiltrate, and numerous *Leishmania*-like amastigotes were observed within the cytoplasm of macrophages (Figure 1). The lymph node exhibited extensive necrosis and abundant macrophages containing numerous *Leishmania*-like amastigotes. Hence, the diagnosis was infectious colitis and adenitis in favor of visceral leishmaniasis; specific stains for fungi were not performed. A sternal puncture was conducted to investigate further the diagnosis, and microscopy examination of the marrow aspirate revealed hemophagocytosis and histiocytes containing intracellular inclusions with a parasitic appearance, suggesting parasitic invasion by *Leishmania*. Consequently, treatment with intramuscular meglumine antimonate was initiated at a dose of 20 mg/kg/day. After 15 days of injections, due to elevated liver enzymes, meglumine antimonate was suspended, and it was replaced with conventional amphotericin B (powder for 50 mg solution) with a dose of 4 mg/kg for 18 days. Additionally, the bone marrow aspirate was sent to a private laboratory to detect *Leishmania* DNA using conventional PCR; after 12 days, the PCR result was negative.

**Figure 2:**
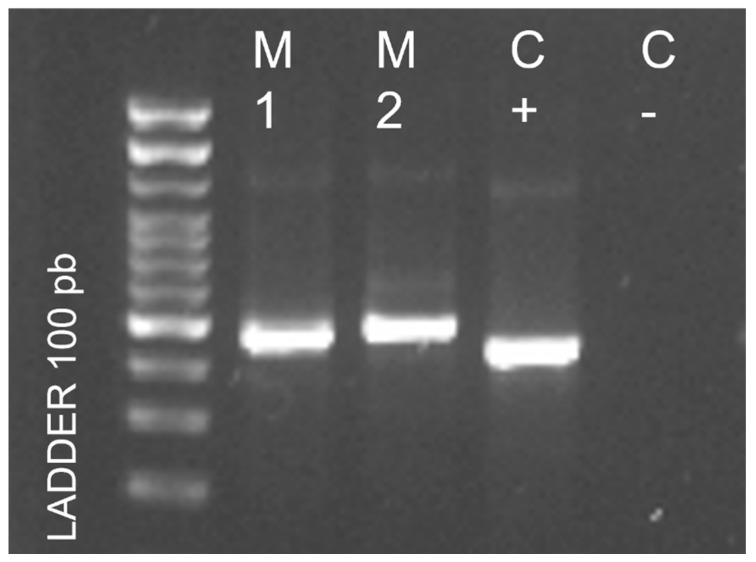
Photography of a 1.0% agarose gel electrophoresis stained with SYBRSafe. The patient’s samples (M1 and M2) show bands between 550 and 600 bp. The positive control (C+) consists of a previously confirmed *H. capsulatum* isolate, validated by sequencing. A 100 bp molecular marker is included.

**Figure 3:**
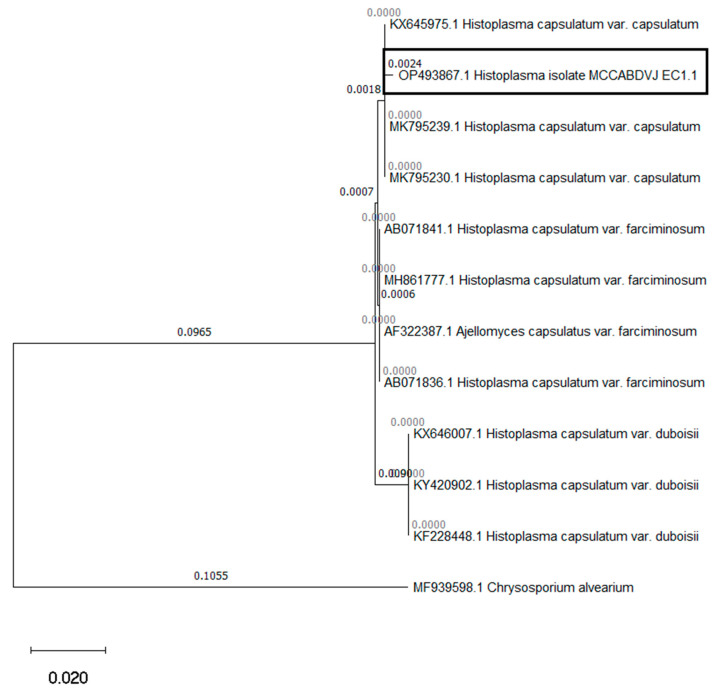
Phylogenetic tree generated using the MEGA 11 software, constructed with the neighbor-joining method, and bootstrap analysis with 1000 replicates. Branch lengths (numeric values on branches) were calculated based on the Kimura’s 2-parameter model with 30 treatment sites and uniform rates. The patient’s isolated sample (accession number OP493867.1) exhibits a close phylogenetic relationship with *H. capsulatum* var. *capsulatum*, showing an evolutionary distance of zero-base substitutions per site. *Chrysosporium alvearium* was used as an outgroup for comparison.

At 4 months of hospitalization, the infant’s condition worsened, manifesting more frequent rectal bleeding, necessitating multiple blood transfusions and resulting in caloric-protein malnutrition. To establish a molecular diagnosis, new bone marrow samples and large intestine biopsies were analyzed at the molecular biology laboratories of the University of the Americas, Quito. Total DNA extraction was performed, followed by end-point PCR using universal primers for fungal identification, ITS1 (5′TCCGTAGGTGAACCTGCGG3′) and ITS4 (5′TCCTCCGCTTATTGATATGC3′), targeting the internal transcribed spacer 1 (ITS) and 5.8S ribosomal RNA ITS2 (https://www.ncbi.nlm.nih.gov/pmc/articles/PMC2812305/pdf/1750-09.pdf, accessed on 1 March 2023). Gel electrophoresis revealed the amplification of a 580 bp band (Figure 2). Sanger sequencing of the amplified product yielded a sequence of approximately 560 bp. The obtained sequence was identified through BLAST analysis against the sequences available in the GenBank database, resulting in a 99.28% sequence identity and 100% coverage percentage, confirming the presence of *H. capsulatum*. A phylogenetic tree was constructed and compared with sequences from GenBank, ultimately identifying the strain as *H. capsulatum* var. *capsulatum* (Figure 3). The GenBank accession number for the sequence is OP493867.1. Sadly, 20 days later, the infant passed away. Written informed consent was obtained from the father for the publication of this case.

## 3. Discussion

This is the first reported case in Ecuador of histoplasmosis diagnosed molecularly by PCR and confirmed as *Histoplasma capsulatum* var. *capsulatum* through DNA sequencing. The microscopic resemblance between *Histoplasma* spp. yeasts and the amastigote forms of *Leishmania* spp. observed in histopathology and cytology led to an incorrect diagnosis and treatment as visceral leishmaniasis (VL). The delayed diagnosis of intestinal and subsequently disseminated histoplasmosis contributed to disease progression, ultimately leading to the patient’s demise. Is important to disseminate this information among healthcare professionals, pathologists, and laboratory workers to ensure accurate and timely clinical and laboratory diagnosis of histoplasmosis.

The microscopic confusion observed in this case, mistaking *H. capsulatum* yeasts with *Leishmania* amastigotes, is likely due to the endemicity of leishmaniasis in tropical regions of Ecuador [30]. However, visceral leishmaniasis (VL) has not been diagnosed in this country but in neighboring countries such as Colombia, Peru, and Bolivia [33]. Microscopy of histopathological and cytological samples of *H. capsulatum* yeasts requires differentiation from several other microorganisms, including protozoa—*Leishmania* spp., *Trypanosoma cruzi*, and *Toxoplasma gondii*—as well as yeasts of other fungi—*Cryptococcus* spp., *Paracoccidioides brasiliensis*, *Coccidioides* spp., *Candida* spp., and *Pneumocystis jirovecii* [28]. The three protozoa stain with H&E, leading to misidentification as *Histoplasma* yeasts, as occurred in this case; these protozoa do not stain with GMS or PAS stains [28]. In this particular case, GMS or PAS stains were not performed initially, as the diagnosis of *Leishmania* amastigotes was made based on H&E staining. In Ecuador, it is crucial to recognize and differentiate these three protozoal infections, since they are also endemic and coexist in tropical regions [30].

Although detecting circulating *Histoplasma* antigens in urine using ELISA has shown high sensitivity (95%), its availability and distribution in endemic countries and regions, including Ecuador, are limited [26,27,28,31]. *Histoplasma* culture was also unavailable at the hospital mentioned. Given the advantages of speed, high sensitivity, and specificity offered by molecular diagnosis using DNA probes, these techniques are widely performed in clinical laboratories in developed countries [1,4], enabling identification of isolates [29]. Therefore, we recommend implementing molecular diagnostic methods in endemic countries such as Ecuador. These techniques provide a fast and reliable diagnosis [31], can be performed on various fluids and tissues, minimize delays in initiating specific treatment, and thus help to prevent disease dissemination and fatalities.

In Ecuador, specific *Histoplasma* varieties have not been identified, and all cases are reported as *H. capsulatum*. It is important to note that identifying the different varieties of *H. capsulatum* genus is significant regarding their geographical distribution, clinical presentation, and microscopy characteristics. *H. capsulatum* var. *capsulatum* is widely distributed and can cause several clinical forms of the disease. On the other hand, *H. capsulatum* var. *duboisii* is exclusively found in Africa and is associated with aggressive disease presentation and characterized by larger yeast forms. Additionally, *H. capsulatum* var. *farciminosum* is found only in Asia and primarily affects horses [1,3]. Although there may be a subdivision of *H. capsulatum* var. *capsulatum* into different clades or species in the Americas, these distinctions do not have clinical implications regarding disease presentation or treatment strategies [1].

In our case, the initial therapeutic management was empirical, targeting bacterial intestinal infections due to fever and diarrhea. Subsequently, treatment was shifted to meglumine antimonate for VL based on the microscopic confusion observed. It is important to note that intestinal histoplasmosis is a common condition characterized by pain, bleeding, perforation, and malabsorption, often accompanied by ulcerations [26]. Unfortunately, in the present case, administering corticosteroids and immunosuppressants for Crohn’s disease, and malnutrition further aggravated the condition and led to disseminated histoplasmosis.

For the treatment of disseminated histoplasmosis, liposomal amphotericin B is recommended as the drug of choice [27]. However, it is unfortunate that this medication is not available in Ecuador due to its high cost, official institutions do not procure it. Additionally, the administration of fluconazole, in this case, was inappropriate, as it has not been shown to be effective against *H. capsulatum* [24]. To prevent similar situations in the future, it is highly recommended to raise awareness among clinical physicians and provide training in diagnostic techniques to laboratory workers. Implementing molecular diagnostic methods in laboratories located in endemic regions and reference hospitals is encouraged. Moreover, antigen test, although can cross react with other fungi, it is highly sensitive and quick.

## Data Availability

In the hospital archive, available on reasonable request.
